# A Thirteen-Gene Expression Signature Predicts Survival of Patients with Pancreatic Cancer and Identifies New Genes of Interest

**DOI:** 10.1371/journal.pone.0105631

**Published:** 2014-09-02

**Authors:** Timothy E. Newhook, Edik M. Blais, James M. Lindberg, Sara J. Adair, Wenjun Xin, Jae K. Lee, Jason A. Papin, J. Thomas Parsons, Todd W. Bauer

**Affiliations:** 1 Department of Surgery, University of Virginia, Charlottesville, Virginia, United States of America; 2 Department of Biomedical Engineering, University of Virginia, Charlottesville, Virginia, United States of America; 3 Department of Public Health Sciences, University of Virginia, Charlottesville, Virginia, United States of America; 4 Department of Microbiology, Immunology, and Cancer Biology, University of Virginia, Charlottesville, Virginia, United States of America; University of Barcelona, Spain

## Abstract

**Background:**

Currently, prognostication for pancreatic ductal adenocarcinoma (PDAC) is based upon a coarse clinical staging system. Thus, more accurate prognostic tests are needed for PDAC patients to aid treatment decisions.

**Methods and Findings:**

Affymetrix gene expression profiling was carried out on 15 human PDAC tumors and from the data we identified a 13-gene expression signature (risk score) that correlated with patient survival. The gene expression risk score was then independently validated using published gene expression data and survival data for an additional 101 patients with pancreatic cancer. Patients with high-risk scores had significantly higher risk of death compared to patients with low-risk scores (HR 2.27, p = 0.002). When the 13-gene score was combined with lymph node status the risk-score further discriminated the length of patient survival time (p<0.001). Patients with a high-risk score had poor survival independent of nodal status; however, nodal status increased predictability for survival in patients with a low-risk gene signature score (low-risk N1 vs. low-risk N0: HR = 2.0, p = 0.002). While AJCC stage correlated with patient survival (p = 0.03), the 13-gene score was superior at predicting survival. Of the 13 genes comprising the predictive model, four have been shown to be important in PDAC, six are unreported in PDAC but important in other cancers, and three are unreported in any cancer.

**Conclusions:**

We identified a 13-gene expression signature that predicts survival of PDAC patients and could prove useful for making treatment decisions. This risk score should be evaluated prospectively in clinical trials for prognostication and for predicting response to chemotherapy. Investigation of new genes identified in our model may lead to novel therapeutic targets.

## Introduction

Pancreatic ductal adenocarcinoma (PDAC) has the shortest survival duration of any solid organ malignancy [1], [2]. Currently, prognostication for patients with PDAC is based on the 7^th^ edition of the American Joint Committee on Cancer (AJCC) staging system that takes into account size and invasive properties of the tumor and presence of nodal and distant metastatic disease [3]. This staging system remains the primary consideration by physicians to determine appropriate treatment as well as offer prognostic information for patients and families [3]. Significant ranges in survival exist within individual AJCC clinical stages [4], [5], [6]; for instance, stage IV patients may live only a few weeks after diagnosis or may live longer than one to two years with treatment. It is likely that this intra-stage variance is due to heterogeneous tumor gene expression resulting in differences in tumor biology.

We report the identification and validation of a 13-gene expression signature that predicts survival for patients with PDAC with stratification of patients into high- and low-risk groups based on the coordinate expression of genes defined by the gene expression signature. Assessment of lymph node status further added to the prognostic efficacy of the signature. The genes and pathways whose expression makes up the 13-gene signature represent possible targets for further research into the biology of PDAC tumors.

## Methods

### Ethics Statement

PDAC sample collection and processing were carried out with approval of the Institutional Review Board of the University of Virginia in coordination with the Biorepository and Tissue Research Facility. All patients provided written consent for participation. This study was carried out in strict accordance with the recommendations in the Guide for the Care and Use of Laboratory Animals of the National Institutes of Health [7]. The protocol was approved by the Animal Care and Use Committee of the University of Virginia (PHS Assurance #A3245-01).

### Propagation of Patient-Derived Tumors in Immunocompromised Mice for Gene Expression Profiling

The collection, pathologic examination, and propagation of human patient-derived PDAC specimens in immunocompromised mice were performed as previously described [8], [9]. Following surgical resection and pathological review of the patient tumor, residual tumor tissues were collected and placed in Roswell Park Memorial Institute media (RPMI) for surgical transplantation in mice. Six to eight week old, male, non-obese, diabetic, severe combined immunodeficient (NOD SCID) and athymic nude mice (National Cancer Institute, Fredricksburg, MD) were used. To achieve more efficient engraftment during initial establishment of the human PDAC tumor line, NOD SCID mice were used for the first two generations. For propagation of the tumor line beyond these first two generations, athymic nude mice were used, as they retain innate immunity (natural killer cells, B lymphocytes, antigen presenting cells, and complement activity), which is impaired in NOD SCID mice. Mice were housed in pathogen-free conditions, acclimated to their new surroundings for at least 48 hours prior to tumor engraftment, and maintained in accordance with institutional standards. All animal surgery was performed under 2,2,2-tribromoethanol anesthesia (4 mg/10 gm body weight). Post-surgery mice were administered ketoprofen 0.1 mg for pain control and were observed continuously for signs of pain or distress (hypoactivity, restlessness, vocalization, hiding, lack of grooming, abnormal posture, tremor, or respiratory distress) until they recovered from anesthesia, then monitored daily for 48 hr for signs of pain or distress. Humane endpoints were observed throughout experiments with mice being sacrificed when tumors reached a volume greater than 1500 mm^3^ by MRI assessment or when mice developed 15% weight loss. Mice were sacrificed via isofluorane anesthesia followed by cervical dislocation.

Human tumors were surgically implanted onto the pancreata of mice immediately following resection from either a patient or earlier generation xenograft. A 1.5-cm left flank incision was used to access the peritoneum of anesthetized mice, the pancreas was exteriorized using a sterile cotton swab and a small piece (∼25 mm^3^) of fresh patient tumor was sutured onto the pancreas using 5-0 Prolene (Ethicon, Cornelia, GA). The pancreas was repositioned and the wound closed using 4-0 Vicryl suture (Ethicon).

Human tumor tissue comprising tumor and associated stroma (without laser microdissection) was preserved after harvest from individual xenografts using AllProtect (Qiagen, Valencia, CA) for efficient RNA preservation. Tissue homogenization was performed utilizing the TissueLyzer LT (Qiagen) and RNA extraction was performed using the RNAeasy kit (Qiagen), according the manufacturer's instructions. Gene expression analysis using the Affymetrix GeneChip platform (Affymetrix, Santa Clara, CA) using the Human Genome U133 Plus 2.0 Arrays and the GeneChipt 3′ IVT Express Labeling Assay was carried out by the University of Virginia Biomolecular Research Facility.

### Development of Prognostic Gene Signature and Statistical Analysis

**Figure 1:**
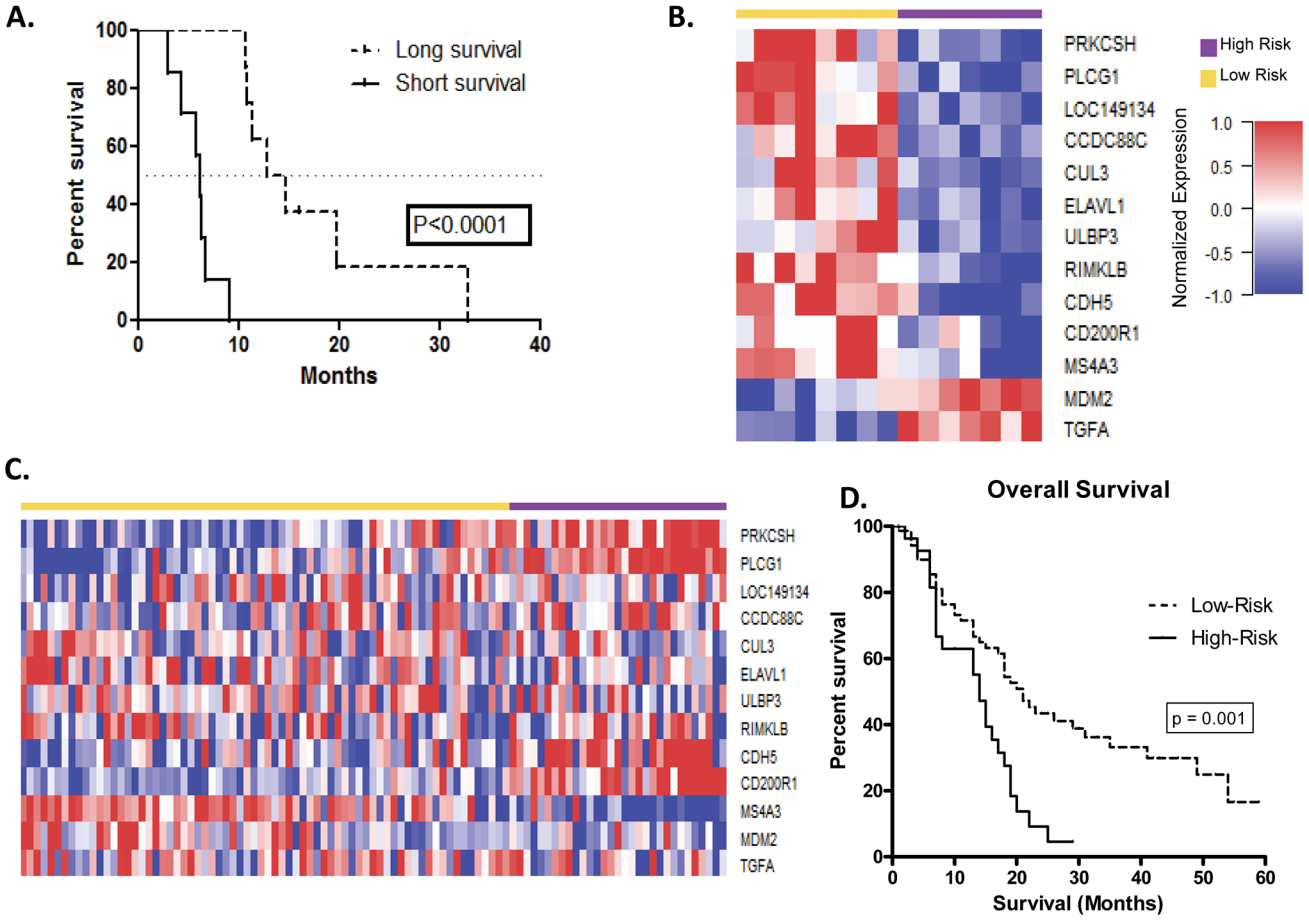
Application of a 13-gene prognostic signature for patients with PDAC. (**A**) Kaplan-Meier overall survival of patients comprising the UVA-15 derivation set grouped by short survival time (n = 7, survival range: 2.0–9.0 mo; median: 6.1 mo) and long survival time (n = 8, survival range: 10.6–32.8 mo; median: 13.7 mo; log-rank p<0.001). (**B**) Expression of 13-genes in the 15-tumor derivation set of patients with PDAC reveals clustering into high- (purple bar) and low-risk (yellow bar) populations. (**C**) Application of the 13-gene signature to an independent validation set of 101 patients with localized and resected PDAC reveals clustering into high- (purple bar) and low-risk (yellow bar) groups based on gene expression. (**D**) Kaplan-Meier overall survival of the independent validation set according to high- and low-risk groups as determined by 13-gene signature (log-rank p = 0.001).

Expression data sets from two distinctive patient cohorts were employed for gene prediction modeling and independent validation (Table 1). The first data set was derived from a cohort of 15 patients with pancreatic cancer at the University of Virginia (UVA-15; GSE46385) and was used for the gene expression biomarker discovery and prediction modeling for patient survival. To identify an appropriate external validation data set, we conducted a search for linked gene expression and overall survival data for patients with pancreatic cancer in the following publicly available databases: National Center for Biotechnology Information – Gene Expression Omnibus (NCBI GEO; http://www.ncbi.nlm.nih.gov/geo/), European Molecular Biology Laboratory – European Bioinformatics Institute Array Express (EBML EBI; https://www.ebi.ac.uk/arrayexpress/), The Cancer Genome Atlas (http://cancergenome.nih.gov/), and Oncomine (https://www.oncomine.org/resource/login.html). From this search, we found a data set derived from a cohort of 101 PDAC patients (Stratford-101; NCBI GEO Database: GSE21501). The second external data set reported appropriate survival information of 45 PDAC patient samples (GSE28735), so the larger external dataset (Stratford-101; GSE21501) was selected as the validation set. Raw expression data were downloaded from GEO, examined for quality control, and pre-processed using robust multi-array averaging (RMA) and quantile normalization methods in the R/Bioconductor programming environment. Patients were grouped by overall survival duration into short survival time (n = 7, survival range: 2.0–9.0 mo; median: 6.1 mo) and long survival time (n = 8, survival range: 10.6–32.8 mo; median: 13.7 mo, Fig. 1A). Using the UVA-15 data set, genes that were significantly differentially expressed between short and long survival groups were identified using both non-parametric Wilcoxon test and two-sample t-test to identify genes that were consistently associated with patient survival. The data were fitted to a Cox proportional hazard regression model using the metagene signatures (principal components) of 15 genes based on a statistical dimension reduction technique.

**Table 1:** Patient, tumor, and treatment data in derivation and validation sets.

*Demographics*	*Derivation Set (n = 15)*	*Validation Set (n = 101)*
**T Stage**		
**1**	1 (7%)	2 (2%)
**2**	6 (40%)	15 (15%)
**3**	8 (53%)	79 (78%)
**4**	0 (0%)	1 (1%)
**Tx**	0 (0%)	4 (4%)
**N Stage**		
**0**	1 (7%)	28 (28%)
**1**	9 (60%)	73 (72%)
**Nx**	5 (33%)	0 (0%)
**M Stage**		
**0**	9 (60%)	101 (100%)
**1**	6 (40%)	0 (0%)
**Overall Stage**		
**Ia**	0 (0%)	1 (1.0%)
**Ib**	1 (7%)	6 (6%)
**IIa**	0 (0%)	19 (20%)
**IIb**	8 (53%)	70 (69%)
**III**	0 (0%)	1 (1%)
**IV**	6 (40%)	0 (0%)
**N/A**	0 (0%)	4 (4%)
**Postoperative Chemotherapy**		
**Adjuvant Gemcitabine (Stage I–III)**		
**No**	0 (0%)	N/A
**Yes**	9 (60%)	N/A
**Palliative Gemcitabine (Stage IV)**		
**No**	1 (7%)	N/A
**Yes**	5 (33%)	N/A
**Median Overall Survival Time (mo)**	10.6±7.6	14.5±13.7

N/A, data not available.

Applying the fitted Cox regression model independently, survival times for patients in the Stratford-101 cohort were determined. The predicted survival times of the Stratford-101 patients were ranked and converted into percentiles - 1 for the patient with the shortest survival time and 100 for the patient with the longest survival time. The statistical significance of the predicted survival scores were evaluated compared to actual patient survival times using Student two-sample t-test at the optimal prediction (percentile) score cutoff that maximized the survival benefit with the highest positive predictive value for long-term survivors. Kaplan-Meier survival analysis was also performed at this cutoff point. Risk hazard ratios were also obtained from the Cox regression model for several contrasting conditions of interest on the Stratford-101 cohort.

Pathway-level gene expression changes between high risk and low risk patients from the Stratford-101 dataset were identified using gene set enrichment analysis. Annotated gene sets for Kyoto Encyclopedia of Genes and Genomes (KEGG) pathways were downloaded from the Molecular Signatures Database [10], [11], [12]. Expression changes for genes and KEGG pathways were evaluated using linear models for microarrays (limma) and gene set variability analysis [13], [14], [15]. A p-value cutoff of 0.05 was applied after false discovery rate (FDR) correction.

## Results

### Patient and tumor characteristics

Table 1 summarizes the information for the 15 patients comprising the derivation set all of whom underwent surgery for PDAC at the University of Virginia. Tumor stage ranged from I to IV, with the majority of patients having stage IIb disease with positive lymph nodes. The 40% of patients in the derivation set with stage IV disease underwent excision/biopsy of a metastasis (4 patients with liver metastases, one patient with peritoneal metastasis, and one with pleural metastasis), but did not undergo resection of their primary tumor. No patients in the derivation set received any form of neoadjuvant therapy, while all patients with localized disease who underwent resection received post-operative gemcitabine-based chemotherapy and 5 of 6 patients with metastatic disease received post-operative palliative gemcitabine-based chemotherapy (Table 1).

A total of 101 patients with localized, resected PDAC comprised the validation set for the gene expression signature [16]. The majority of patients within this group had stage IIb PDAC (72%) and none had stage IV disease, as all tumors were resectable (Table 1). Frequency of neoadjuvant and adjuvant therapies administered to this patient group was unavailable.

### Identification and validation of a 13-gene prognostic signature

The 13 genes that comprise the 13-gene expression signature are described in Table 2. To evaluate the predictive ability of the initial candidate 13-gene prognostic signature we assessed an independent gene expression data set derived from 101 patients with localized primary PDAC [16]. The optimal cutoff point differentiating low risk score vs. high risk score was determined to be 70 by maximizing the Youden index (sensitivity+specificity−1) with the constraint that the proportion of high-risk patients was at least 10% or higher for practical clinical applications. The 13-gene prognostic score was then applied with a cutpoint of 70 (e.g. low risk: <70; high risk: >70), which provided the significant survival benefit and difference between patients with low vs. high risk scores in the derivation set of 15 patients (Fig. 1A). Heat maps of the gene expression signature for the UVA-15 derivation set and 101-tumor validation set are shown in Figure 1. This application of the gene signature effectively stratified patients into high- and low-risk groups with a median overall survival (MS) of 14.0 v. 21.0 months, respectively (Fig. 1D). Furthermore, patients in the high-risk group had a greater than two-fold increase risk of death as compared to those in the low-risk group (HR 2.27 [95% CI 1.34–3.85], p = 0.002; Fig. 1D).

**Table 2:** Characteristics and reported findings for the genes comprising the 13-gene prognostic signature.

*Gene*	*Probe Set ID*	*Name*	*Function*	*Role in Pancreatic Cancer*	*Role in Other Cancers*
***CCDC88C***	231288_at	Coiled-coil domain containing 88c	Negative regulator of Wnt pathway signaling	Not reported	14q32 SNP associated with ER+ breast cancer in AA females [37].
***CD200R1***	1552875_a_at	CD200 Receptor- 1	Down-regulation of myeloid-lineage proliferation	Not reported	Implicated in immune-evasion and metastasis of SCC [38]. Ligand-receptor interaction implicated in AML, MM [45], [46].
***CDH5***	204677_at	Cadherin 5, type 2	Cell-cell adhesion molecule	Upregulated in VHL-associated pancreatic neuroendocrine tumors [47].	Highly expressed in invasive breast cancer [48]. Mutated in advanced triple-negative breast cancers [49].
***CUL3***	201372_s_at	Cullin 3	Polyubiquitination and component of E3 ubiquitin protein ligase complex	Not reported	Associated with aggressiveness and prognosis in invasive bladder cancer [39]. Implicated in glioblastoma tumorigenesis [40].
***FAM80B***	242870_at	Ribosomal modification protein rimK-like family member B	N-acetylaspartylglutamate synthetase 1 (NAAGS-1)	Not reported	Not reported
***ELAVL1***	244660_at	ELAV (embryonic lethal, abnormal vision, drosophila)-like; HuR	RNA-binding protein	7-fold increase in mortality with low levels, and modulates gemcitabine efficacy [33].	Associated with prognosis, growth, and invasion in breast cancer [50], [51]. Expression correlates with tumor stage in cervical cancer and high grade NSCLC [52], [53].
***LOC149134***	230541_at	Uncharacterized locus	Uncharacterized locus on chromosome 1	Not reported	Not reported
***MDM2***	225160_x_at	E3 ubiquitin protein ligase	Feedback regulator of p53 signaling	Overexpressed in PDAC [34]. Expressed in Ras-dependent manner in cell lines [36].	Altered in many cancers, including glioblastoma, lipsarcoma, melanoma, and breast cancer [54].
***MS4A3***	210254_at	Membrane-spanning 4-domains, subfamily A, member 3	Cell-cycle modulator	Highly expressed in pancreatic adenocarcinoma [35].	Highly expressed in multiple cancers, including seminomas, endometrium, ovary, and breast [35].
***PLCG1***	202789_at	Phospholipase C, gamma 1	Cell-surface receptor signal transduction	Not Reported	Associated with increased motility and invasion of HER2-amplified breast cancer cell lines [55]. Increased in primary breast cancers [56].
***PRKCSH***	214080_x_at	Protein kinase C substrate 80K-H	B-subunit of glucosidase II, substrate of protein kinase C	Not reported	Not reported
***TGFA***	205015_s_at	Transforming growth factor alpha	Growth and proliferation	Overexpression induced PDAC and precursor lesions in mice along with *KRAS* overexpression [32].	Expression associated with survival in expression profiles of glioblastoma [57]. Expression correlated with poor survival in esophageal cancer [13].
***ULBP3***	231748_at	UL16 binding protein 3	Stress-induced ligand of NK cell activation	Not reported	Increased expression correlates with improved survival in patients with B-CLL [58].

SNP, single-nucleotide polymorphism; ER, estrogen receptor; AA, African American; SCC, squamous cell carcinoma; AML, acute myeloid leukemia; MM, multiple myeloma; VHL, von Hippel-Lindau; NSCLC, non-small cell lung carcinoma; NK, natural killer; B-CLL, B-cell chronic lymphocytic leukemia.

### A 13-gene expression signature more accurately predicts survival of PDAC patients when combined with nodal status

**Figure 2:**
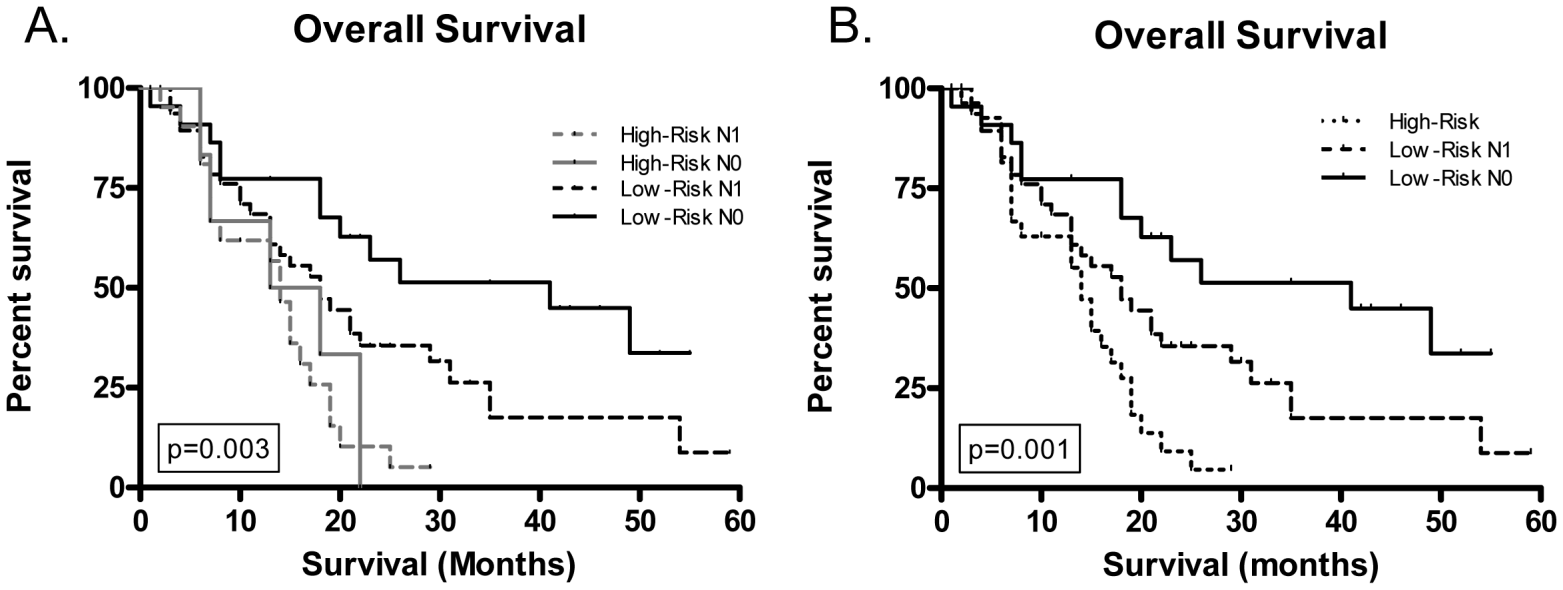
A 13-gene expression signature combined with lymph node status accurately predicts patient survival. Kaplan-Meier overall survival of (**A**) a validation set of 101 patients with localized, resected PDAC according to 13-gene prognostic score combined with pathologic lymph node status at the time of surgery, and (**B**) the same 101 patients grouped according to either high-risk 13-gene prognostic score alone or low-risk 13-gene prognostic score plus pathologic nodal status at time of surgery.

We next sought to further refine our prognostic gene score by incorporating lymph node status with the risk score. This effectively stratified patients into four groups – low-risk score, node-negative (n = 22); low-risk score, node-positive (n = 48); high-risk score, node-negative (n = 6); and high-risk score, node-positive (n = 25). As demonstrated in Figure 2A, patients in the low-risk, node-negative group had the best prognosis (MS: 41.0 mo), followed by low-risk, node-positive patients (MS: 18.0 mo). Patients with high-risk score had poor median survival, independent of lymph node status (node-negative: 15.5 mo; node-positive: 14.0 mo; p = NS; Fig. 2A). Compared to low risk, node-negative patients, the high-risk, node-positive patients had a near 4-fold increased risk of death (HR = 3.77 [95%CI: 1.75–8.10], p = 0.007), and high-risk, node-negative patients had 3-fold increase (HR = 3.09 [95% CI: 1.05–9.03], p = 0.007), whereas low-risk, node-positive patients had a 2-fold increased risk (HR = 1.95 [95% CI: 0.98–3.88], p = 0.007; Fig. 2A).

Interestingly, what is apparent from the aforementioned results is that patients with high-risk tumors based on gene expression have a poor prognosis, regardless of nodal status; whereas, nodal status further refines prognosis for patients with low-risk tumors. Thus, there are three distinct prognostic groups: high-risk patients (n = 31; MS: 14.0 mo; Fig. 2B), low-risk, node-positive patients (n = 48; MS: 18.0 mo; Fig. 2B), and low-risk, node-negative patients (n = 22; MS: 41.0 mo; 2B).

### Pathway analysis reveals key pathways differentially expressed between tumors with high- and low-risk prognostic signatures

To link the observed changes in gene expression with molecular and cellular pathways that may impact in the observed differential survival between high- and low-risk groups, we evaluated 5199 of 17623 genes and 97 of 186 KEGG pathways that were significantly differentially expressed between patients with high-risk and low-risk prognostic scores. Differentially expressed KEGG pathways between high- and low-risk patients included *cancer cell signaling pathways* (MAPK, VEGF, MTOR, and ERBB signaling pathways) and *cancer pathways* (acute myeloid leukemia, non-small cell lung cancer, chronic myeloid leukemia, and pancreatic cancer; Table 3). Additionally, three genes from the 13-gene prognostic signature, *MDM2*, *PLCG1*, and *TGFA*, were represented in 9 out of the top 20 significant pathways. These results revealed that genes involved in canonical cancer signaling pathways were most differentially expressed between high-risk and low-risk tumors and that the activity of these pathways may be responsible for the observed difference in survival between high- and low-risk patient groups.

**Table 3:** Top 20 KEGG pathways significantly enriched (p<0.05) for upregulation in high risk patients relative to low risk patients.

KEGG Pathway	Prognostic Genes*	FDR
Acute Myeloid Leukemia		1.22E-09
Fc Gamma Receptor-Mediated Phagocytosis	PLCG1	3.84E-07
Non-Small Cell Lung Cancer	PLCG1, TGFA	3.84E-07
Chronic Myeloid Leukemia	MDM2	5.75E-07
Neurotrophin Signaling Pathway	PLCG1	1.94E-06
Vascular Smooth Muscle Contraction		2.54E-06
B Cell Receptor Signaling Pathway		2.63E-06
ERBB Signaling Pathway	PLCG1, TGFA	3.16E-06
Chemokine Signaling Pathway		3.16E-06
Glioma	MDM2, PLCG1, TGFA	3.52E-06
Dilated Cardiomyopathy		3.52E-06
T-Cell Receptor Signaling Pathway	PLCG1	5.61E-06
MAPK Signaling Pathway		6.35E-06
GNRH Signaling Pathway		6.35E-06
Insulin Signaling Pathway		7.18E-06
Prion Diseases		7.4E-06
Pancreatic Cancer	TGFA	7.51E-06
MTOR Signaling Pathway		9.99E-06
Long Term Depression		9.99E-06
VEGF Signaling Pathway	PLCG1	1.49E-05

*Pathways that include genes from the prognostic signature are shown.

FDR, False Discovery Rate; KEGG, Kyoto Encyclopedia for Genes and Genomes.

## Discussion

We report a 13-gene expression signature, derived from gene expression analysis of 15 patients with PDAC and externally validated on gene expression data from a separate cohort of 101 patients, which accurately predicts patient survival. Because this model was based on overall survival for patients with stage I to IV disease, we believe this is the most logical and accurate prognostic gene expression signature reported for patients with PDAC.

Due to variation in survival within AJCC clinical stages and to the large genomic heterogeneity within PDAC tumors, investigation into prognostic gene expression patterns has been increasingly reported [3], [16], [17], [18], [19], [20]. In a previously reported study of patients with metastatic versus non-metastatic PDAC, a 6-gene prognostic signature correlated with survival; however, this signature was derived from tumor stage at presentation and not from patient survival [16]. Moreover, no overlap exists between the candidate genes in the 6-gene signature and the 13-gene expression signature described herein, which was in fact based on patient survival. Thus, because of the selection of the derivation patient set, we believe this 13-gene expression signature outperforms others reported in the literature for patients with PDAC.

Commercially available mutational and gene expression profiling platforms are increasingly utilized as adjuncts to conventional clinical treatment algorithms for treatment of cancers including breast, prostate, and colon cancer [21], [22], [23], [24]. These expression analyses are arguably most robust in predicting outcomes for patients with breast cancer, including OncotypeDX and MammaPrint [25], [26]. These platforms are used to predict early outcomes and risk of metastasis in breast cancer; however, further applications of these tools helps tailor treatment based on predicting response to therapies [25], [27], [28], [29], [30], [31]. To date, no such prognostic tool is commercially available for patients with PDAC; however, predicting survival for patients with PDAC based on individual tumor biology would clearly benefit patients' and clinicians' therapeutic decisions.

The individual genes whose expression levels were used to derive this 13-gene prognostic signature reveal an intriguing network of pathways that impact PDAC patient survival (Table 2 and 3). Many of these genes have been implicated in various human cancers, including pancreatic cancer; however, some have not been reported to be associated with any cancers to date. Recognizable genes such as *TGFA*, *ELAVL1* and *MDM2*, and less so *MS4A3* are over-expressed in PDAC lesions or associated with patient prognosis [32], [33], [34], [35], [36]. Interestingly, genes such as *CCDC88C*, *CD200R1*, and *CUL3* have been associated with prognosis or being highly expressed in other forms of cancer, however they have not been reported in PDAC to the best of our knowledge [37], [38], [39], [40]. Their involvement in the 13-gene prognostic signature is the first report of their expression being implicated in patient survival in PDAC. The identification of measureable differences in gene expression between PDAC tumors from patients with varying survival times supports the further application of our gene signature and investigation into these various pathways.

No patients within the derivation set received neoadjuvant therapy of any form, and therefore the gene expression analysis of these tumor samples represents the profile of the tumor prior to any systemic therapy (Table 1). However, within the prognostic calculation is the expression of *ELAVL1*, also known as Hu antigen-R (HuR), which has been implicated in PDAC response to chemotherapy [33]. In fact, PDAC patients with low levels of *ELAVL1* expression have a 7-fold increase in mortality [33]. In our analysis, high-risk PDAC tumors have a decreased expression of *ELAVL1* as compared to low-risk tumors and 93% of patients within the derivation set received standard-of-care adjuvant or palliative gemcitabine-based therapy. Unfortunately, clinical data on adjuvant therapy regimen of the 101 patients within validation set was unavailable; however, it stands to reason that the majority of patients also received standard of care gemcitabine adjuvant therapy. The 13-gene expression signature may predict patient response to adjuvant gemcitabine therapy and we plan to evaluate the ability of the 13-gene signature to predict response to therapy in future studies.

A particular strength of this study is that the patient tumors within the derivation set are from a good representative sample of AJCC disease stages (Table 1). A limitation of our study is that the validation set was comprised of 101 patients with localized, resectable PDAC and thus the great majority of patients had AJCC stage IIb or less (Table 1). We hypothesize that having an increased number of patients with stage III or IV disease in the derivation set would only serve to increase the stratification based on our gene signature with and without the addition of nodal status because this would add a larger number of patients with either high-risk disease, or, low-risk node-positive disease and push these survival stratifications toward greater statistical significance. Despite this, the fact that our signature was externally validated on a set of 101 patients with PDAC whose gene expression and clinical data was publically available adds to the unbiased nature of our study.

Due to the coarse nature of the current clinical PDAC staging paradigm, additional prognostic tools are needed to aid in therapeutic decision-making. The decision to undergo pancreatic resection, which is the only potentially curative option for PDAC patients, is a stressful one considering the 15–26% readmission rate and complications occurring in approximately 40% of patients after surgery even at centers of excellence [41], [42], [43], [44]. Additionally, systemic chemotherapy can be associated with significant toxicity and can negatively impact quality of life. Given the uncertain benefit of chemotherapy for a given patient, the concerns over treatment toxicity and quality of life are key factors facing patients. The ability to offer patients and clinicians accurate prognostic data about PDAC tumors based on gene expression measurement of individual tumor biology is invaluable and could influence the decision to offer (or forego) therapy. This prognostic instrument has the potential to aid patients and physicians in making treatment decisions, which ultimately may affect outcome and impact quality of life. Future evaluation of this gene signature will assess for the ability to predict response to chemotherapy.
